# Loss of expression and prognosis value of alpha-internexin in gastroenteropancreatic neuroendocrine neoplasm

**DOI:** 10.1186/s12885-018-4449-8

**Published:** 2018-06-26

**Authors:** Yuhong Wang, Yuanjia Chen, Xiaoxing Li, Wanming Hu, Yu Zhang, Luohai Chen, Minhu Chen, Jie Chen

**Affiliations:** 1 Department of Gastroenterology, The First Affiliated Hospital, Sun Yat-sen University, No.58 Zhongshan II Road, Yuexiu District, Guangzhou, 510080 China; 2 Department of Gastroenterology, Peking Union Medical College Hospital, Beijing, China; 3 State Key Laboratory of Oncology in South China, Collaborative Innovation Center for Cancer Medicine, Sun Yat-sen University Cancer Center, Guangzhou, China; 4 Department of Pathology, Sun Yat-sen University Cancer Center, Guangzhou, China

**Keywords:** Gastroenteropancreatic neuroendocrine neoplasm. Alpha-internexin. Expression. Prognosis

## Abstract

**Background:**

The neuronal intermediate filament alpha-internexin (α-internexin) is a cytoskeleton protein which is involved in the tumor initiation and progression. In this study, we examined the expression and prognosis value of α-internexin in gastroenteropancreatic neuroendocrine neoplasms (GEP-NENs).

**Methods:**

α-internexin was detected with immunohistochemical staining in 286 tumor specimens from patients with GEP-NENs. Methylation status of *α-internexin* was evaluated by bisulfite genomic sequencing. We assessed the prognostic value of α-internexin and its correlation with relevant clinicalpathological characteristics.

**Results:**

The reduced/loss of expression rate of α-internexin in GEP-NEN was 73.4% (210/286), while the positive expression rate was 26.6% (76/286). The difference of α-internexin deficiency was not statistically significant between gastrointestinal NENs (GI-NENs) and pancreatic NENs (pNENs). However, we found significant difference of reduced/loss of α-internexin expression among different sites of GI-NENs (χ^2^ = 43.470, *P* < 0.001). The reduced/loss of expression of α-internexin was significantly associated with poorly differentiation (*P* < 0.001) and advanced tumor stage (*P* < 0.001). Univariate analyses showed that reduced/loss of expression of α-internexin predicted worse overall survival (OS) in GEP-NEN patients (*P* < 0.001), especially in subtype of GI-NENs (*P* < 0.001). However, in multivariable regression analysis, α-internexin expression was not an independent prognostic factor. The hypermethylation of *α-internexin* gene was significantly correlated with protein deficiency in GI-NENs, but not in pNENs. Hypermethylation of several CpG sites was significantly associated with poorly differentiated and advanced stage (*P* values range from 0.018 to 0.044). However, the methylation status of *α-internexin* was not associated with patient OS.

**Conclusions:**

The expression of α-internexin was highly heterougeneous in different sites of GEP-NENs. The reduced/loss of expression of α-internexin was closely related to tumors with aggressiveness and patient’s adverse prognosis. The hypermethylation of the regulatory region examined may be an important epigenetic regulation mechanism of α-internexin deficiency in subtype of GI-NENs.

**Electronic supplementary material:**

The online version of this article (10.1186/s12885-018-4449-8) contains supplementary material, which is available to authorized users.

## Background

Gastroenteropancreatic neuroendocrine neoplasms (GEP-NENs), which originate from neuroendocrine cells distributed throughout the digestive system, comprise a heterogeneous family with wide and complex clinical behaviors. They are often associated with a very aggressive clinical course and 60%~ 80% of NENs are metastatic when identified, although being generally more indolent than carcinomas [1]. At present, only few reliable molecular biomarkers could predict the biological behavior and prognosis of the patients with GEP-NEN [2, 3]. Therefore, searching for novel biomarkers is an important issue on GEP-NEN.

Alpha-internexin is a 66-kDa type IV intermediate filament protein. As a cytoskeleton protein, previous studies showed that it is mainly expressed in various kinds of central and peripheral neurons from early development [4], and is frequently detected in medulloblastomas [5] and neuroblastomas which shared some common features with neuroendocrine tumors [6]. Previous several studies have detected the expression of α-internexin on particular types of GEP-NENs such as well-differentiated endocrine tumors, or a single site of tumors (pancreas, small intestinal, appendix or rectum). These studies reported the expression of α-internexin varied from different sites of GEP-NEN. They also made inconsistent conclusions on the relationship between α-internexin expression and tumor biological behavior [7–9]. Therefore, the expression of α-internexin and its clinical and prognosis value in GEP-NEN is worth of investigation.

In the current study, we determined the expression of α-internexin in a large cohort of GEP-NEN using immunohistochemistry and findings were associated with clinicopathological variables and patient prognosis. We further investigated the regulation of the epigenetic mechanisms of *α-internexin* gene expression, and explored the clinical and prognostic role of *α-internexin* methylation in GEP-NEN. In addition, previous studies revealed that GEP-NEN is a type of tumor with marked heterogeneity. Tumors originated from gastrointestinal tract may considerably differ from those from pancreas [1]. Therefore, the analyses were performed not only in GEP-NEN as a whole, but also in gastrointestinal NENs (GI-NENs) and pancreatic NENs (pNENs) as separate subgroup in this study.

## Methods

### Patients information

A total of 286 patients with histologically confirmed sporadic GEP-NEN in The First Affiliated Hospital, Sun Yat-sen University from September 2002 to December 2014 were enrolled in the study to determine the expression of α-internexin. The methylation status of *α-internexin* was evaluated by bisulfite genomic sequencing (BGS) in 116 cases out of 286 patients. Patients’ clinicopathologic characters are summarized in Table 1 and Table 2.

**Table 1:** Clinicopathological characteristics of patients with α-internexin immunohistochemical detection

Demographic and Clinical Characteristics		N	%
GEP-NENs (*n* = 286)			
Sex	Male	173	60.5
	Female	113	39.5
Age (years) at diagnosis	≤50	134	46.9
	> 50	152	53.1
	Median (range)	53 (16–85)	
Functional status	Nonfunctional	234	81.8
	Functional	52	18.2
	Insulinoma	42	14.7
	Vasoactive intestinal polypeptidoma	7	2.4
	Carcinoid syndrome	1	0.3
	Somatostatinoma	1	0.3
	Gastrinoma	1	0.3
Tumor location	Gastrointestinal tract	162	56.6
	Rectum	60	21.0
	Stomach	43	15.0
	Duodenum	21	7.3
	Esophagus	18	6.3
	Jejunum/ileum	9	3.1
	Appendix	6	2.1
	Colon	5	1.7
	Pancreas	93	32.5
	Other	31	10.8
	Metastasis of unknown primary	25	8.7
	Biliary tract	5	1.7
	Greater omentum	1	0.3
Tumor grade^a^	G1	120	43.2
	G2	57	20.5
	G3	101	36.3
Tumor type^a^	NET	180	64.7
	NET G1	120	43.2
	NET G2	57	20.5
	NET G3	3	1.1
	NEC	91	32.7
	MANEC	7	2.5
Tumor stage	I	79	27.6
	II	61	21.3
	III	45	15.7
	IV	101	35.3
GI-NENs (*n* = 162)			
Tumor grade^b^	G1	60	38.5
	G2	21	13.5
	G3	75	48.1
Tumor type^b^	NET	81	51.9
	NET G1	60	38.5
	NET G2	21	13.5
	NEC	70	44.9
	MANEC	5	3.2
Tumor stage	I	48	29.6
	II	26	16.0
	III	37	22.8
	IV	51	31.5
pNENs (*n* = 93)			
Tumor grade^c^	G1	54	59.3
	G2	27	29.7
	G3	10	11.0
Tumor type^c^	NET	84	92.3
	NET G1	54	59.3
	NET G2	27	29.7
	NET G3	3	3.3
	NEC	7	7.7
	MANEC	0	0
Tumor stage	I	30	32.3
	II	30	32.3
	III	4	4.3
	IV	29	31.2

^a^ 278 cases both for tumor grade and tumor type; ^b^ 156 cases both for tumor grade and tumor type; ^c^ 91cases both for tumor grade and tumor type

*GEP-NEN* Gastroenteropancreatic neuroendocrine neoplasm, *NET* Neuroendocrine tumor, *NEC* Neuroendocrine carcinoma, *MANEC* Mixed adenoneuroendocrine carcinoma, *GI-NEN* Gastrointestinal neuroendocrine neoplasm, *pNEN* Pancreatic neuroendocrine neoplasm

**Table 2:** Clinicopathological characteristics of patients with *α-internexin* methylation

Demographic and Clinical Characteristics		N	%
GEP-NENs (*n* = 116)			
Sex	Male	72	62.1
	Female	44	37.9
Age (years) at diagnosis	≤50	50	43.1
	> 50	66	56.9
	Median (range)	55 (16–83)	
Functional status	Nonfunctional	89	76.7
	Functional	27	23.3
	Insulinoma	26	22.4
	Gastrinoma	1	0.9
Tumor location	Gastrointestinal tract	54	46.6
	Stomach	18	15.5
	Rectum	10	8.6
	Duodenum	10	8.6
	Esophagus	10	8.6
	Colon	4	3.4
	Jejunum/ileum	2	1.7
	Pancreas	49	42.2
	Other	13	11.2
	Metastasis of unknown primary	10	8.6
	Biliary tract	3	2.6
Tumor grade^a^	G1	41	36.3
	G2	23	20.4
	G3	49	43.4
Tumor type^a^	NET	64	56.6
	NET G1	41	36.3
	NET G2	23	20.4
	NEC	43	38.1
	MANEC	6	5.3
Tumor stage	I	27	23.3
	II	35	30.2
	III	28	24.1
	IV	26	22.4
GI-NENs (*n* = 54)			
Tumor grade^b^	G1	8	15.4
	G2	7	13.5
	G3	37	71.2
Tumor type^b^	NET	15	28.8
	NET G1	8	15.4
	NET G2	7	13.5
	NEC	33	63.5
	MANEC	4	7.7
Tumor stage	I	5	9.3
	II	14	25.9
	III	23	42.6
	IV	12	22.2
pNENs (*n* = 49)			
Tumor grade^c^	G1	32	66.7
	G2	11	22.9
	G3	5	10.4
Tumor type^c^	NET	43	89.6
	NET G1	32	66.7
	NET G2	11	22.9
	NEC	5	10.4
	MANEC	0	0
Tumor stage	I	22	44.9
	II	20	40.8
	III	1	2.0
	IV	6	12.2

^a^ 113 cases both for tumor grade and tumor type; ^b^ 52 cases both for tumor grade and tumor type; ^c^ 48 cases both for tumor grade and tumor type

GEP-NEN: Gastroenteropancreatic neuroendocrine neoplasm; NET: Neuroendocrine tumor; NEC: Neuroendocrine carcinoma; MANEC: Mixed adenoneuroendocrine carcinoma; GI-NEN: Gastrointestinal neuroendocrine neoplasm; pNEN: Pancreatic neuroendocrine neoplasm

A functional tumor was defined as overproducing a hormone such as 5-hydroxytryptamine, gastrin, glucagon, insulin, somatostatin and vasoactive intestinal peptide, which causes clinical symptoms. The pathology of each patient was reviewed by a pathologist (Wanming Hu) according to the 4th edition World Health Organization classification of tumors of the digestive system [10]. Tumor-Node-Metastasis (TNM) stage was adopted according to the European Neuroendocrine Tumor Society Consensus Guidelines [11, 12] in tumors originated from the gastrointestinal tract, pancreas and metastatic NENs of unknown primary. Other sites included esophagus, biliary tract were classified by 2017 American Joint Committee on Cancer Staging Atlas 8th edition [13].

### Immunohistochemistry

To detect the expression of α-internexin in GEP-NEN tissues, immunohistochemical studies were performed on paraffin sections using an EnVision method. Sections of tumor specimens (4 μm thick) from formalin-fixed paraffin-embedded were used for immunohistochemical examinations. The slides were dewaxed with xylene, rehydrated in a graded series of ethanol. Heat-induced epitope retrieval was done using a pressure cooker at 1000 W for 2.5 min in preheated Tris-EDTA buffer (pH 9.0). Endogenous peroxidase activity was blocked by incubating the slides in 3% hydrogen peroxide for 20 min at room temperature. The slides were transferred to phosphate-buffered saline and then incubated at 4 °C with rabbit monoclonal anti-α-internexin (1:400; MAB5224; Millipore, Darmstadt, Germany) overnight. In the second day, sections were incubated in secondary antibody (Real EnVision Detection kit, ready-to-use; K5007; Dako; Agilent Technologies, Inc., Santa Clara, CA, USA) for 1 h at room temperature. The substrate chromogen, 3.3′ -diaminobenzidine, enabled visualization of the complex via a brown precipitate. Hematoxylin (blue) counterstaining enabled the visualization of the cell nuclei with a light microscope (4500; Olympus Corporation, Tokyo, Japan). Omission of primary antibody served as a negative control.

### Histological interpretation

The α-internexin positive staining refers to cytoplasm staining to yellow or dark brown. Nonneoplastic cells (lymphocytes, stromal cells, endothelial cells and liver cells) served as an internal positive control in all tissue sections. The criteria [7] of semi-quantitative grading of IHC: (−) means no positive staining in tumor cells; (±) < 20% tumor cells showing positive staining; (+) ≥20% but < 50% tumor cells showing positive staining; (++) ≥50% but < 75% tumor cells shown positive staining; (+++) ≥ 75% tumor cells shown positive staining. We defined < 20% tumor cells with staining of α-internexin as reduced or loss of (reduced/loss of) expression, and otherwise defined as positive. All slides were evaluated independently by Wanming Hu who was blinded to the patients’ clinical data.

### Bisulfite genomic sequencing

Genomic DNA was extracted from 116 GEP-NEN tissues using QIAamp DNA FFPE Tissue Kit (56404; Qiagen, Hilden, Germany) and treated with sodium bisulfite using an EZ DNA Methylation-Gold Kit (D5006; Zymo Research, Orange, CA, USA). The bisulfite-modified DNA was amplified using primer pairs (Forward: 5′- GATTTGGAGAAGAAGGTGGAGT-3′, Reverse: 5′-TGATTGTGGTTAAATTAGAT TTGAT-3′) that specifically amplify the region (+ 683~ + 834) relative to the transcription start site (TSS) of *α-internexin*. A total volume of PCR amplification mixture was 25 μl containing 1 μl DNA, 1 μl of each primer, 12.5 μl Zymo Taq Premix (E2003; Zymo Research, Orange, CA, USA) and 6.5 μl water. PCR was run in Verti Thermal cycler (Applied Biosystems, Foster City, CA, USA). The PCR cycling parameters were as follows: denaturing of 95 °C (10 min); then 42 cycles of 95 °C (30s), 56 °C (40s), 72 °C (40s); a final elongation step of 72 °C (7 min). The target fragment was 152 bp in length containing fifteen CpG sites: GATTTGGAGAAGAA GGTGGAGT**CG**TTGTTGGA**CG**AGTTGGTTTT**CG**TA**CG**TTAGGTGTA**CG**A**CG**AGGAGGTAGT**CG**AGTTGTTGGTTA**CG**TTGTAGG**CG**T**CG**T**CG**TAGGT**CGCG**GT**CG**AGGTGGA**CG**TGATTGTGGTTAAATTAGATTTGAT (Each vertical bar represents a single CpG site). PCR products were sequenced by the BGI Science and Technology, Ltd. (Guangzhou) Research Center. When analyzed, due to the former three CpG sites (included in the + 705~ + 728 region) couldn’t provide exactly methylation level, we analyzed the rest of 12 CpG sites (included in the + 729~ + 834 region) in this study. Each CpG site was recorded as S_1_, S_2_, S_3_…S_12_. Methylation percentage was calculated according to the formula: methylation% = *H*_C_/(*H*_C_ + *H*_T_) × 100% (*H*_C_ = height of peak C and *H*_T_ = height of peak T). Accordingly, average methylation percentage of total 12 CpG sites was calculated by the formula: methylation% = [*H*_C1_/(*H*_C1_ + *H*_T1_) + *H*_C2_/(*H*_C2_ + *H*_T2_)… + *H*_C12_/(*H*_C12_ + *H*_T12_)]/12× 100%.

### Statistical analysis

Statistical analyses were performed using SPSS version 23.0 (IBM, Chicago, IL). Descriptive statistics of qualitative data such as patient’s general data, positive expression rates, were expressed as numbers and percentages. The association of α-internexin expression with various clinicopathologic features was analyzed using Pearson chi-square test. The correlation between *α-internexin* methylation status and α-internexin protein expression level, patient’s clinicopathologic features were estimated by Mann-Whitney or Kruskal-Wallis test. Receiver operating characteristic (ROC) curve was used to estimate the cutoff value of the methylation percentage. Overall survival (OS) analyses were performed using the Kaplan-Meier cureves and log-rank test. Multivariate analyses were performed using Cox proportional hazards regression by including variables that were significantly associated with survival in log-rank test. Hazard ratios (HRs) and 95% confidence intervals (CIs) were calculated. A two-sided *P* value of < 0.05 indicates statistically significance.

## Results

### Immunohistochemical expression of α-internexin in GEP-NEN

**Fig. 1:**
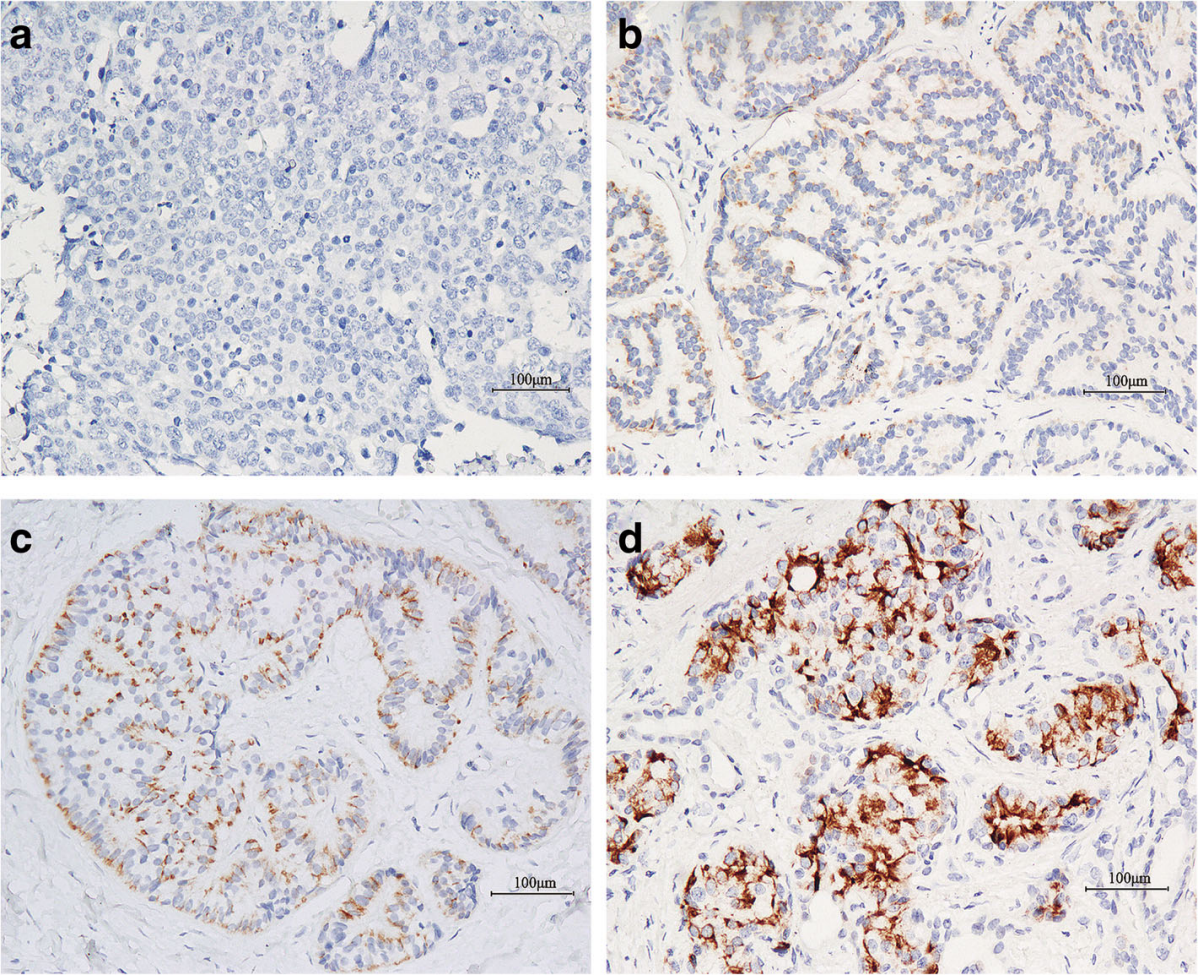
Immunohistochemical staining of α-internexin in gastroenteropancreatic neuroendocrine neoplasm (using the EnVision method). **a** Gastric NEC, G3, negative staining. **b** Rectal NET, G1, weak positive staining. **c** Rectal NET, G1, moderate positive staining. **d** Pancreatic NET, G1, strong positive staining. Magnification, × 20. NET: Neuroendocrine tumor; NEC: Neuroendocrine carcinoma

As shown in Fig. 1, α-internexin was positively immunostained in the cytoplasm of tumor cells, and varied from weak-incomplete to strong-complete. No nuclear immunostaining was observed. The reduced/loss of expression rate of α-internexin was 73.4% (210/286), while the positive expression rate was 26.6% (76/286). The reduced/loss of expression of α-internexin was significantly higher in nonfunctional tumors than in those with hormonal syndrome (76.5% vs. 59.6%; χ^2^ = 6.213, *P* = 0.013). The α-internexin deficiency was not statistically different between GI-NENs and pNENs (76.5% vs. 67.7%; *P* = 0.126). However, different sites in GI-NENs had significant different frequency of α-internexin deficiency (χ^2^ = 43.470, *P* < 0.001). Tumor sites with the highest reduced/loss of expression percentages of α-internexin included esophagus (18/18, 100%) and jejunum/ileum (9/9, 100%), followed by stomach (42/43, 97.7%), duodenum (17/21, 81.0%), colon (4/5, 80.0%), rectum (32/60, 53.3%) and appendix (2/6, 33.3%).

### Correlation of α-internexin expression with tumor grade, type and stage

In patients with GEP-NENs, the reduced/loss of expression of α-internexin in tumors graded as G1, G2 and G3 were 57.5, 63.2 and 98.0%, respectively (χ^2^ = 49.934, *P* < 0.001). In addition, reduced/loss of expression of α-internexin was also significantly higher in poorly differentiated neuroendocrine carcinoma (NEC) and mixed adenoendocrine carcinoma (MANEC) than in well differentiated neuroendocrine tumor (NET) (98.0% vs. 60.0%; χ^2^ = 46.807, *P* < 0.001). The reduced/loss of expression rate of α-internexin in tumors of stage III + IV was 87.0%, which was significantly higher than that of stage I + II (59.3%; χ^2^ = 28.106, *P* < 0.001).

In subtype of GI-NENs, tumors graded as G3 and classified as NEC + MANEC had higher α-internexin reduced/loss of expression percentages than G1, G2 and NET (both *P* < 0.001). α-internexin deficiency was also significantly higher in tumors of stage III + IV than in stage I + II (χ^2^ = 25.786, *P* < 0.001).

In subtype of pNENs, the reduced or loss of expression of α-internexin was associated with advanced stage (χ^2^ = 4.638, *P* = 0.031), but not correlated with tumor grade or tumor type (*P* = 0.231 and *P* = 0.299, respectively).

The correlation of α-internexin expression with patient’s characteristics is summarized in Table 3.

**Table 3:** Association of α-internexin protein expression with clinicopathological variables

Characteristics	N	Reduced/loss of expression (%)	χ2 value	*P* value
GEP-NENs (n = 286)				
Functional status			6.213	0.013
Nonfunctional	234	179(76.5)		
Functional	52	31(59.6)		
Tumor location			2.356	0.308
Gastrointestinal tract	162	124(76.5)	2.340^d^	0.126^d^
Rectum	60	32(53.3)	43.470^e^	< 0.001^e^
Stomach	43	42(97.7)		
Duodenum	21	17(81.0)		
Esophagus	18	18(100)		
Jejunum/ileum	9	9(100)		
Appendix	6	2(33.3)		
Colon	5	4(80.0)		
Pancreas	93	63(67.7)		
Other	31	23(74.2)		
Tumor grade^a^			49.934	< 0.001
G1	120	69(57.5)		
G2	57	36(63.2)		
G3	101	99(98.0)		
Tumor type^a^			46.807	< 0.001
NET	180	108(60.0)		
NEC + MANEC	98	96(98.0)		
Tumor stage			28.106	< 0.001
I + II	140	83(59.3)		
III + IV	146	127(87.0)		
GI-NENs (n = 162)				
Tumor grade^b^			41.938	< 0.001
G1	60	31(51.7)		
G2	21	14(66.7)		
G3	75	74(98.7)		
Tumor type^b^			40.004	< 0.001
NET	81	45(55.6)		
NEC + MANEC	75	74(98.7)		
Tumor stage			25.786	< 0.001
I + II	74	43(58.1)		
III + IV	88	81(92.0)		
pNENs (n = 93)				
Tumor grade^c^			2.929	0.231
G1	54	34(63.0)		
G2	27	19(70.4)		
G3	10	9(90.0)		
Tumor type^c^			1.080	0.299
NET	84	56(66.7)		
NEC + MANEC	7	6(85.7)		
Tumor stage			4.638	0.031
I + II	60	36(60.0)		
III + IV	33	27(81.8)		

^a^ 278 cases both for tumor grade and tumor type; ^b^ 156 cases both for tumor grade and tumor type; ^c^ 91cases both for tumor grade and tumor type; ^d^ The χ2 and *P* value were computed by the contrast between gastrointestinal tract and pancreas; ^e^ The χ2 and *P* value were computed by the contrast among different sites of gastrointestinal tract

GEP-NEN: Gastroenteropancreatic neuroendocrine neoplasm; NET: Neuroendocrine tumor; NEC: Neuroendocrine carcinoma; MANEC: Mixed adenoneuroendocrine carcinoma; GI-NEN: Gastrointestinal neuroendocrine neoplasm; pNEN: Pancreatic neuroendocrine neoplasm

### Correlation of α-internexin expression with overall survival

253 out of 286 patients received long-term follow up with a median duration of 3.59 years (range 0.02–14.6 years). At the last follow-up, 86 patients (34.0%) had died: four died of postoperative complications or other diseases and 82 from tumor progression. Only NEN-related deaths were considered as events for survival analysis.

**Fig. 2:**
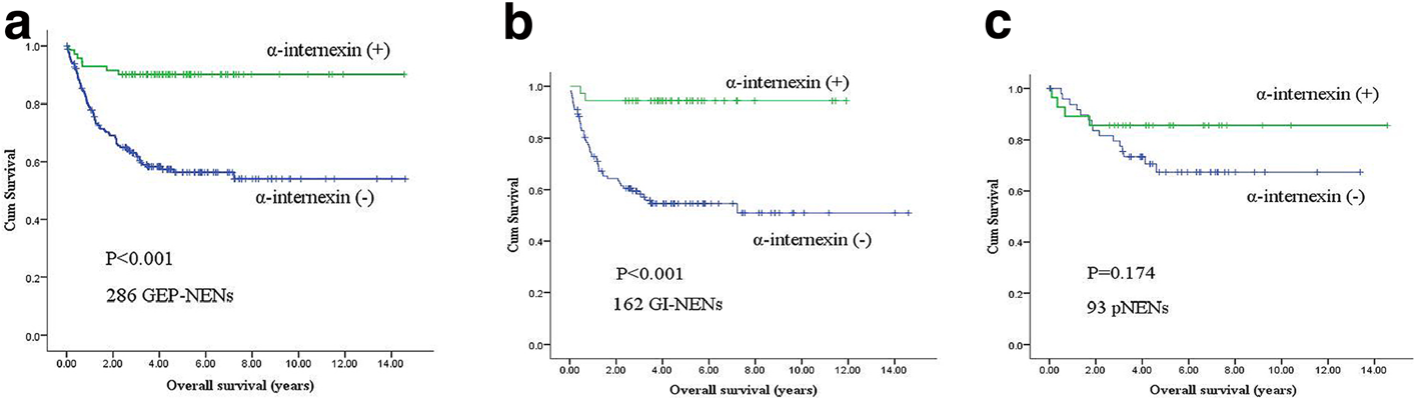
Kaplan-Meier survival curves of patients with GEP-NENs and subtypes of GEP-NENs according to α-internexin expression. **a** Overall survival by α-internexin expression in GEP-NENs. **b** Overall survival by α-internexin expression in GI-NENs. **c** Overall survival by α-internexin expression in pNENs. GEP-NEN: Gastroenteropancreatic neuroendocrine neoplasm; GI-NEN: Gastrointestinal neuroendocrine neoplasm; pNEN: Pancreatic neuroendocrine neoplasm

In patients with GEP-NENs, Kaplan-Meier survival curves showed that the mean overall survival time of patients with reduced/loss of expression of α-internexin was 8.6 years, while those with positive expression was 13.2 years (Fig. 2a; χ^2^ = 21.968, *P* < 0.001). Multivariable analysis demonstrated that α-internexin was not an independent prognostic marker (HR 0.770, 95% CI 0.298–1.985, *P* = 0.588). Expectedly, tumor grade and TNM stage were independently associated with overall survival (*P* = 0.019 and *P* < 0.001, respectively).

GI-NEN patients with reduced/loss of expression of α-internexin had poorer survival than those with positive expression (Fig. 2b; mean OS: 8.2 years vs. 11.3 years; χ^2^ = 16.094, *P* < 0.001). However, multivariable Cox’s model demonstrated that α-internexin was not an independent predictor of survival (HR 1.303, 95% CI 0.241–7.058, *P* = 0.759). α-internexin deficiency was not significantly associated with OS in patients with pNEN (Fig. 2c; χ^2^ = 1.850, *P* = 0.174). Tumor grade, tumor type and TNM stage were independent prognostic factors both in subtype of GI-NENs and pNENs. The results of multivariate Cox proportional hazard model are provided in Table 4.

**Table 4:** Multivariate analysis of overall survival in patients

Factors	GEP-NENs		GI-NENs		pNENs	
	HR (95% CI)	*P* value	HR (95% CI)	*P* value	HR (95% CI)	*P* value
Sex	1.403 (0.815–2.414)	0.222	1.261 (0.638–2.493)	0.505	0.443 (0.145–1.350)	0.152
Age (years) at diagnosis	1.332 (0.753–2.357)	0.325	1.404 (0.763–2.585)	0.275	0.373 (0.081–1.727)	0.207
Functional status	1.048 (0.352–3.120)	0.932	1.300 (0.137–12.375)	0.820	0.657 (0.155–2.789)	0.569
Tumor location	0.776 (0.406–1.484)	0.443	–	–	–	–
Tumor grade	0.106 (0.017–0.640)	0.019	0.070 (0.013–0.389)	0.002	2.473 (1.164–5.103)	0.018
Tumor type	0.468 (0.115–1.903)	0.289	6.531 (2.602–16.392)	< 0.001	1.705 (0.233–12.472)	0.599
Tumor stage	0.201 (0.083–0.489)	< 0.001	3.800 (1.522–9.487)	0.004	21.083 (2.503–177.556)	0.005
α-internexin expression^a^	0.770 (0.298–1.985)	0.588	1.303 (0.241–7.058)	0.759	3.998 (0.935–17.092)	0.062

^a^α-internexin expression means reduced/loss of expression of α-internexin

*GEP-NEN* Gastroenteropancreatic neuroendocrine neoplasm; GI-NEN: Gastrointestinal neuroendocrine neoplasm; pNEN: Pancreatic neuroendocrine neoplasm. *HR* Hazard ratios, *CI* Confidence intervals

### Correlation of *α-internexin* methylation status with its protein expression

**Fig. 3:**
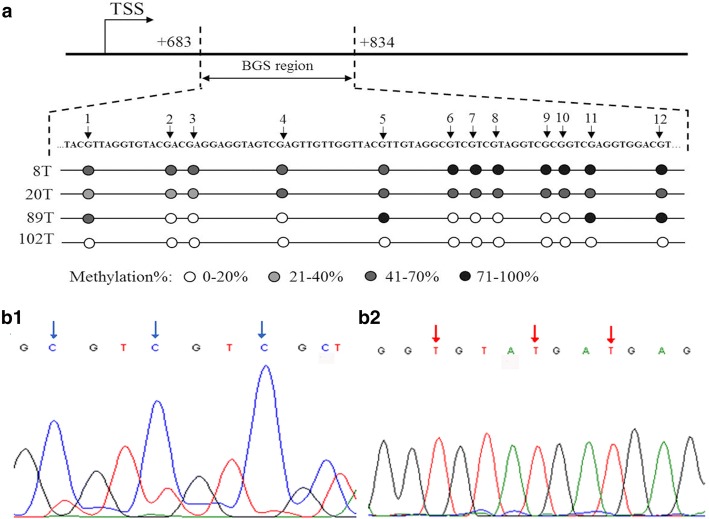
Representative results of bisulfite genome sequencing for methylation analysis. **a** The bisulfite genome sequencing (BGS) analysis was performed on the *α-internexin* genomic region (+ 683~ + 834) relative to the transcription start site (TSS). DNA sequence of the region for BGS and methylation level of CpG sites are shown. **b**1 Pancreas neuroendocrine tumor, G1, α-internexin positive expression, 3 CpG sites were unmethylated (red arrow). **b**2 Rectum neuroendocrine carcinoma, G3, loss expression of α-internexin, 3 CpG sites were methylated (blue arrow)

Methylation status of *α-internexin* was detected by BGS in 116 cases (Fig. 3)The median methylation percentage of total CpG sites was similar between tumors with positive α-internexin expression and those without α-internexin expression (64.1% vs. 65.8%; *P* = 0.091). We further analyzed the correlation of methylation level of each CpG site with α-internexin expression. The methylation level of CpG S_4_ and S_6_ were both significantly higher in tumors with α-internexin reduced/loss of expression than those with positive ones (*P* = 0.015 and *P* = 0.019, respectively). However, methylation levels of other CpG sites were not associated with α-internexin expression (*P* > 0.05).

In subtype of GI-NENs, the median methylation percentage of total CpG sites was higher in tumors with α-internexin deficiency than that in tumors with α-internexin expression (68.5% vs. 61.8%; *P* = 0.011). The methylation level of each CpG site was also significantly higher in tumors with α-internexin protein deficiency (*P* values range from 0.002 to 0.039). In subtype of pNENs, no associations between methylation levels of *α-internexin* and protein expression were observed (the average of total 12 CpG sites as well as each site were all examined) (*P* > 0.05). Major results of correlation between *α-internexin* methylation status and protein expression are showed in Table 5. Full results are listed in Additional file 1: Table S1.

**Table 5:** Correlation of *α-internexin* methylation status with protein expression (major results)

	Methylation levels, % Median (range)		Z value	*P* value
	α-internexin (−)	α-internexin (+)		
GEP-NENs (n = 116)				
Average of total 12 CpG sites	65.8 (0–85.3)	64.1 (0–79.5)	1.692	0.091
S_4_	63.3 (0–80.8)	57.8 (0–73.5)	2.424	0.015
S_6_	66.0 (0–89.3)	61.0 (0–81.4)	2.338	0.019
GI-NENs (n = 54)				
Average of total 12 CpG sites	68.5 (0–81.7)	61.8 (11.5–67.8)	2.539	0.011
S_1_	53.2 (0–100)	45.8 (12.6–61.8)	2.062	0.039
S_2_	53.5 (0–68.3)	41.6 (0–55.3)	2.855	0.004
S_3_	50.0 (0–67.3)	41.3 (0–52.6)	2.633	0.008
S_4_	64.6 (0–79.5)	56.7 (0–65.6)	2.897	0.004
S_5_	65.6 (0–85.7)	62.1 (11.5–76.3)	2.094	0.036
S_6_	67.4 (0–89.3)	56.0 (0–68.0)	3.130	0.002
S_7_	66.0 (0–85.7)	61.0 (0–69.4)	2.105	0.035
S_8_	82.9 (0–93.8)	70.3 (0–84.1)	2.583	0.010
S_9_	66.2 (0–84.4)	54.9 (0–67.7)	2.695	0.007
S_10_	80.5 (0–93.6)	65.4 (0–81.6)	2.606	0.009
S_11_	83.2 (0–96.9)	76.0 (10.8–83.3)	2.517	0.012
S_12_	78.6 (0–88.2)	66.6 (13.2–79.0)	2.595	0.009
pNENs (n = 49)				
Average of total 12 CpG sites	63.6 (49.1–85.3)	66.0 (24.0–79.5)	0.164	0.870

S_1_, S_2_...S_12_ means each CpG site in the region (+ 729~ + 834) of *α-internexin*

*GEP-NEN* Gastroenteropancreatic neuroendocrine neoplasm, *GI-NEN* Gastrointestinal neuroendocrine neoplasm; pNEN: Pancreatic neuroendocrine neoplasm

#### Correlation of *α-internexin* methylation status with clinicopathological variables

In patients with GEP-NENs, there were no correlation between *α-internexin* methylation and clinicopathological features, such as tumor functional status, tumor location, tumor grade, tumor type and TNM stage. Similar results were also found in subtype of pNENs.

In subtype of GI-NENs, methylation level of total 12 CpG sites was significantly higher in tumors of stage III + IV than that in stage I + II (68.4% vs. 61.7%; χ^2^ = 5.847, *P* = 0.016). Furthermore, methylation level of CpG S_8_ was significantly associated with tumor grade (*P* = 0.033). Methylation levels of CpG S_2_~S_4_, S_6_ and S_8_ were also significantly associated with tumor type (*P* values range from 0.014 to 0.036). Methylation levels of either site in CpG S_1_~ S_5_, S_10_ and S_11_ were significantly higher in advanced stage tumors (*P* values range from 0.018 to 0.044). The association between clinicopathological features and *α-internexin* methylation status in patients with GEP-NENs, subtype of GI-NENs and pNENs are listed in Additional file 2: Table S2.

#### Correlation of *α-internexin* methylation status with overall survival

A total of 116 patients with *α-internexin* methylation detection received long-term follow up with a median duration of 3.53 years (range, 0.04–11.92 years). At the final follow-up, 30 patients (25.9%) had succumbed to the disease.

**Fig. 4:**
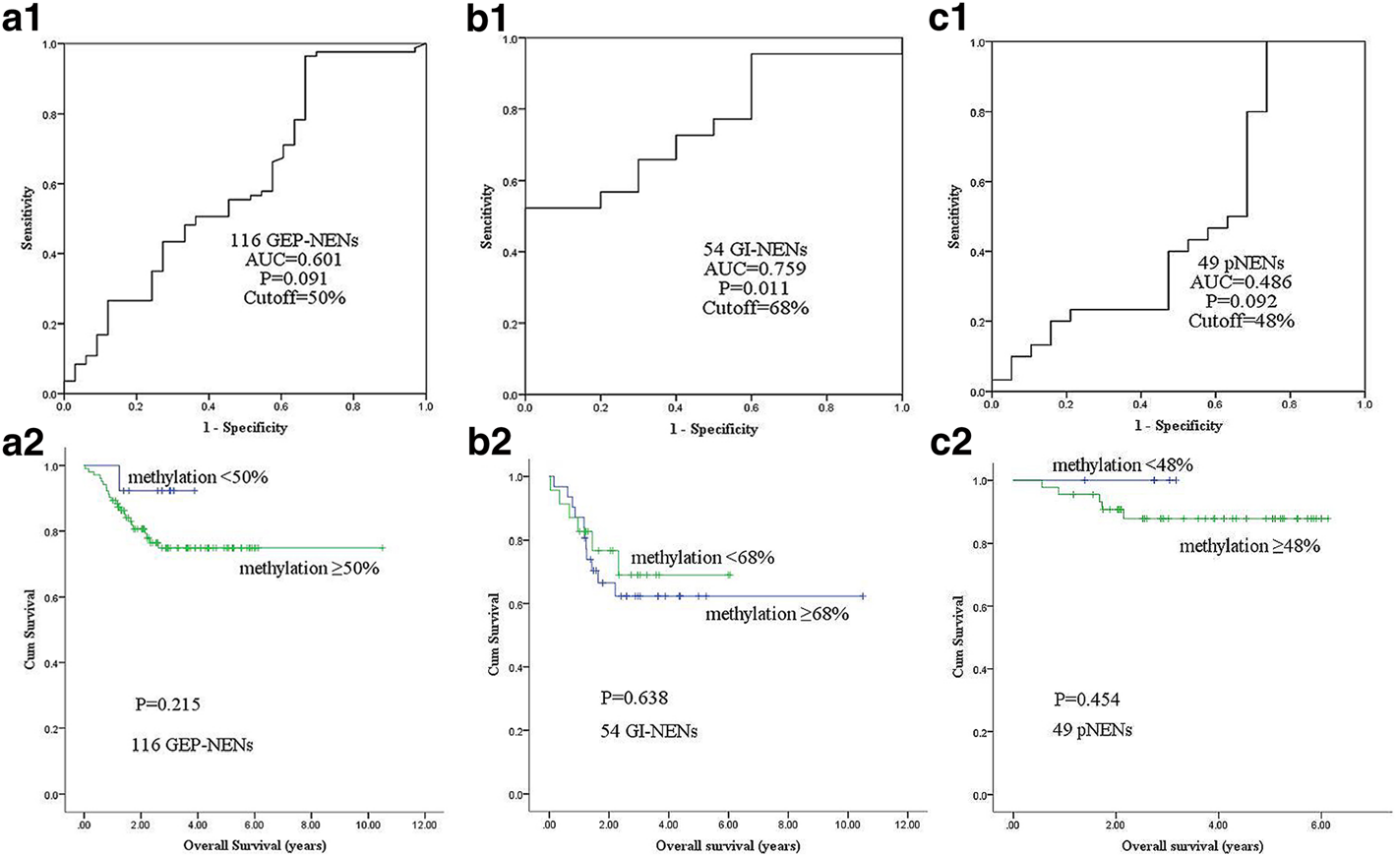
Correlation of *α-internexin* methylation with overall survival. **a**1, **b**1, **c**1 Receiver operating characteristic (ROC) curve was used to determine a best cutoff value to define the methylation status of *α-internexin* in GEP-NENs, subtype of GI-NENs and pNENs. **a**2, **b**2, **c**2 Overall survival by *α-internexin* methylation (results were examined by methylation level of the average of total 12 CpG sites) in GEP-NENs, subtype of GI-NENs and pNENs. ROC: Receiver operating characteristic; AUC: Area under ROC curve; GEP-NEN: Gastroenteropancreatic neuroendocrine neoplasm; GI-NEN: Gastrointestinal neuroendocrine neoplasm; pNEN: Pancreatic neuroendocrine neoplasm

In patients with GEP-NENs, first ROC curve was performed to achieve the suitable cutoff value (50%), to define the methylation status of the examined region (Fig. 4a1). Patients were divided into higher (≥50%) and lower (< 50%) *α-internexin* methylation level groups. Kaplan-Meier analysis showed that no significantly statistical difference was found between *α-internexin* methylation and patient survival. In subtype of GI-NENs and pNENs, univariate analyses also failed to reveal a significant association between *α-internexin* methylation and tumor-related death. ROC curve and major results are showed in Fig. 4. Full results are listed in Additional file 3: Table S3.

## Discussion

As a cytoskeleton protein, several lines of evidence have suggested that α-internexin may play roles in the cell differentiation, the composition and development of intermediate filaments cytoskeleton, as well as in the tumor initiation and progression [4]. Previous studies showed that α-internexin was frequently detected in medulloblastomas and neuroblastomas. The deficiency of α-internexin was confirmed to be associated with malignant biological behaviors [5, 6, 14]. Until now, we know only little about the expression of α-internexin in GEP-NEN according to several previous studies. A small sample study revealed that the α-internexin expression rate was 100% (12/12) in appendiceal well-differentiated NEN, while 50% (4/8) in rectal cases [8]. Liu B et al. detected 350 cases of pNEN showed that the reduced/loss of expression of α-internexin was 46.6%. In nonfunctional pNENs, the reduced/loss of α-internexin expression was much higher than that in functional pNENs (66% vs. 32.5%, *P* = 5.78 × 10^− 10^) [7]. However, these studies only focused on a single site of tumors (appendix, rectum or pancreas). In this study, we systematically examined the expression of α-internexin in 286 cases of GEP-NEN tissues. We found that overall α-internexin deficiency rate was 73.4%. The reduced/loss of α-internexin expression was significantly increased in tumors without hormonal syndrome, which was comparable to the results reported by Liu B. Furthermore, α-internexin deficiency percentage was slightly higher in GI-NENs (76.5%, 124/162) compared with pNENs. The highest deficiency percentages of sites were the esophagus (18/18, 100%) and jejunum/ileum (9/9, 100%), followed by stomach (42/43, 97.7%), duodenum (17/21, 81.0%), colon (4/5, 80.0%), rectum (32/60, 53.3%) and appendix (2/6, 33.3%), with significantly difference (χ^2^ = 43.470, *P* < 0.001). Our results showed that α-internexin expression demonstrates marked heterogeneity and differences in tumor sites.

Modlin et al. enrolled 50 pNENs and 42 SI-NENs showed that α-internexin expression were significantly higher in G2 tumors and metastasis than G1 and primaries [9]. In contrast with the above results, Liu B et al. revealed that the reduced expression of α-internexin was significantly higher in metastasis (71.8% vs. 34.5%; *P* = 1.97 × 10^− 10^). Tumors with G3 (only 9 cases) showed a higher α-internexin deficiency rate (the reduced/loss of α-internexin expression of tumors with G1, G2 and G3 were 47.3, 51.5 and 77.8%, respectively), although no statistically significant difference was observed (*P* = 0.213) [7]. However, these previous studies mainly focused on well-differentiated endocrine tumors (G1 and G2). In the present study, in agreement with Liu B et al. ‘s findings, our results revealed that a gradual decline in the reduced/loss of α-internexin expression in poorly- (tumors graded as G3 or classified as NEC + MANEC) and well-differentiated tumors (G1, G2 or NET; both *P* < 0.001). In addition, α-internexin expression deficiency was significantly higher in tumors of stage III + IV than stage I + II (87.0% vs. 59.3%; *P* < 0.001). Similar results were found in subtype of GI-NENs. In subtype of pNENs, although no significant correlation was observed between α-internexin expression and tumor differentiation, α-internexin deficiency was higher in tumors with advanced stage (*P* = 0.031). Therefore, the current study indicated that the reduced/loss of α-internexin was significantly associated with tumor differentiation and stage, a decrease in α-internexin expression with increasing malignancy in GEP-NEN.

Previous studies showed that expression of α-internexin was correlated with better overall survival in gliomas [15, 16]. Their findings are consistent with our observations that α-internexin positive expression is a favorable prognostic marker. In our study, we found that the reduced/loss of expression of α-internexin was significantly associated with a shorter survival time not only in GEP-NEN patients, but also in subtype of GI-NENs in univariate analyses, suggesting that α-internexin deficiency was significantly associated with a shortened survival time of patients. However, multivariable analysis demonstrated that α-internexin was not an independent prognostic marker. In pNEN patients, Liu B et al. found that reduced/loss of expression of α-internexin predicted worse survival [7], which was inconsistent with our study. In the present study, although no statistically difference, we observed a tendency towards poorer survival in pNEN patients with α-internexin deficiency (mean OS: 9.8 vs. 12.6 years; *P* = 0.174). These findings suggested that patients with α-internexin deficiency had a worse prognosis in GEP-NENs.

It has been well-documented that hypermethylation of CpG islands in gene promoter could induce the transcriptional silencing of the gene [17–19]. Liu B et al. detected the methylation status of *α-internexin* promoter (− 107~ + 96 region) by denaturing high-performance liquid chromatography in 17 pNENs and 8 paired tissues, found that hypomethylation of *α-internexin* gene was associated with protein expression in vivo (*P* = 0.015) [7]. In contrast with the previous study, bisulfite sequencing of the + 683~ + 834 region of *α-internexin* gene was examined in our study. Although the correlation of gene methylation level of total CpG sites with α-internexin expression was not statistically significant in our GEP-NEN cohort, further analysis with each CpG site found that two CpG sites (S_4_ and S_6_) showed higher methylation levels in tumors without α-internexin expression. Importantly, in subtype of GI-NENs, hypermethylation of this region was closely related with reduced/loss of expression of α-internexin. The result was confirmed not only in the examination of total CpG sites but also in each examined site. Furthermore, several CpG sites including S_1_~ S_6_, S_8_ S_10_ and S_11_ had higher methylation levels in tumors with poorly differentiated and advanced stage. However, in subtype of pNENs, we found that *α-internexin* methylation was not associated with protein expression, and clinicopathological features, such as tumor functional status, grade, type and TNM stage. Therefore, we speculated that the regulatory region (+ 683~ + 834) may be crucial for regulating α-internexin expression in GI-NENs, but not in pNENs. Previous study also reported a strong negative correlation between methylation and gene expression was found in downstream of the promoter up to 8 kb away [20], supporting our findings. In consequence, further demethylation studies are required to validate the role of the examined region in the regulatory of *α-internexin* expression. Moreover, we found hypermethylation of most CpG sites in the region suggested more malignancy in GI-NEN.

Gene methylation has been reported as a promising predictive biomarker in many human cancers [21]. So far, the contribution of epigenetic changes to the prognosis of GEN-NENs is still largely unknown. In our study, we explored the prognostic value of *α-internexin* methylation in GEP-NEN for the first time. We found a tendency towards shorter survival in patients with higher methylation level of *α-internexin*. Similar results were also found in subtype of GI-NENs and pNENs. In GEP-NEN, the value of *α-internexin* inactivation by gene methylation deserves further investigation. The prognostic role of *α-internexin* methylation needs to be validated in systematically, prospective studies with a larger sample size.

## Conclusions

The expression of α-internexin was highly heterougeneous in different sites of GEP-NENs. The reduced/loss of expression of α-internexin was closely related to tumors with aggressiveness and patient’s adverse prognosis. The hypermethylation of the regulatory region examined may be an important epigenetic regulation mechanism of α-internexin deficiency in subtype of GI-NENs.

## Additional files

Additional file 1 **Table S1.** Correlation of *α-internexin* methylation status with protein expression. (DOCX 27 kb)

Additional file 2 **Table S2.** Correlation of *α-internexin* methylation status with clinicopathological variables. (DOCX 49 kb)

Additional file 3 **Table S3.** Correlation of *α-internexin* methylation status with overall survival. (DOCX 28 kb)

## Abbreviations

BGS: Bisulfite genomic sequencing
CI: Confidence intervals
GEP-NEN: Gastroenteropancreatic neuroendocrine neoplasm
GI-NEN: Gastrointestinal neuroendocrine neoplasm
HR: Hazard ratios
MANEC: Mixed adenoendocrine carcinoma
NEC: Neuroendocrine carcinoma
NET: Neuroendocrine tumor
OS: Overall survival
pNEN: Pancreatic neuroendocrine neoplasm
ROC: Receiver operating characteristic
TNM: Tumor-Node-Metastasis
TSS: Transcription starting site
α-internexin: Alpha-internexin
