# Supplementary material for: Loss of expression and prognosis value of alpha-internexin in gastroenteropancreatic neuroendocrine neoplasm
Source: BMC Cancer. 2018 Jun 26;18:691. doi: 10.1186/s12885-018-4449-8 (PMC6020194; doi:10.1186/s12885-018-4449-8)
Supplement: Supplementary file 1 — Table S1. Correlation of α-internexin methylation status with protein expression. (DOCX 27 kb) [file 12885_2018_4449_MOESM1_ESM.docx]

**Supplementary Table 1** Correlation of *α-internexin* methylation status with protein expression

|  | Methylation levels, % Median (range) | | Z value | *P* value |
| --- | --- | --- | --- | --- |
|  | α-internexin (-) | α-internexin (+) |  |  |
| **GEP-NENs (n=116)** |  |  |  |  |
| Average of total 12 CpG sites | 65.8 (0-85.3) | 64.1 (0-79.5) | 1.692 | 0.091 |
| S_1_ | 50.0 (0-100) | 50.5 (0-66.7) | 1.320 | 0.187 |
| S_2_ | 50.0 (0-76.1) | 50.0 (0-69.0) | 1.467 | 0.142 |
| S_3_ | 50.0 (0-98.9) | 50.0 (0-67.3) | 1.442 | 0.149 |
| S_4_ | 63.3 (0-80.8) | 57.8 (0-73.5) | 2.424 | 0.015 |
| S_5_ | 64.4 (0-87.5) | 62.5 (0-78.6) | 1.619 | 0.106 |
| S_6_ | 66.0 (0-89.3) | 61.0 (0-81.4) | 2.338 | 0.019 |
| S_7_ | 62.0 (0-87.0) | 61.7 (0-79.0) | 1.769 | 0.077 |
| S_8_ | 82.4 (0-96.8) | 79.2 (0-90.3) | 1.781 | 0.075 |
| S_9_ | 64.0 (0-84.3) | 61.4 (0-78.7) | 1.867 | 0.062 |
| S_10_ | 78.9 (0-93.6) | 76.6 (0-88.5) | 1.640 | 0.101 |
| S_11_ | 82.1 (0-96.9) | 78.3 (0-92.3) | 1.833 | 0.067 |
| S_12_ | 75.4 (0-100) | 72.3 (0-89.3) | 1.778 | 0.075 |
| **GI-NENs (n=54)** |  |  |  |  |
| Average of total 12 CpG sites | 68.5 (0-81.7) | 61.8 (11.5-67.8) | 2.539 | 0.011 |
| S_1_ | 53.2 (0-100) | 45.8 (12.6-61.8) | 2.062 | 0.039 |
| S_2_ | 53.5 (0-68.3) | 41.6 (0-55.3) | 2.855 | 0.004 |
| S_3_ | 50.0 (0-67.3) | 41.3 (0-52.6) | 2.633 | 0.008 |
| S_4_ | 64.6 (0-79.5) | 56.7 (0-65.6) | 2.897 | 0.004 |
| S_5_ | 65.6 (0-85.7) | 62.1 (11.5-76.3) | 2.094 | 0.036 |
| S_6_ | 67.4 (0-89.3) | 56.0 (0-68.0) | 3.130 | 0.002 |
| S_7_ | 66.0 (0-85.7) | 61.0 (0-69.4) | 2.105 | 0.035 |
| S_8_ | 82.9 (0-93.8) | 70.3 (0-84.1) | 2.583 | 0.010 |
| S_9_ | 66.2 (0-84.4) | 54.9 (0-67.7) | 2.695 | 0.007 |
| S_10_ | 80.5 (0-93.6) | 65.4 (0-81.6) | 2.606 | 0.009 |
| S_11_ | 83.2 (0-96.9) | 76.0 (10.8-83.3) | 2.517 | 0.012 |
| S_12_ | 78.6 (0-88.2) | 66.6 (13.2-79.0) | 2.595 | 0.009 |
| **pNENs (n=49)** |  |  |  |  |
| Average of total 12 CpG sites | 63.6 (49.1-85.3) | 66.0 (24.0-79.5) | 0.164 | 0.870 |
| S_1_ | 47.4 (38.1-80.6) | 52.7 (0-66.7) | 0.351 | 0.725 |
| S_2_ | 47.2 (36.1-76.1) | 53.2 (0-69.0) | 0.452 | 0.652 |
| S_3_ | 48.7 (29.3-98.9) | 53.4 (0-67.3) | 0.246 | 0.805 |
| S_4_ | 62.9 (43.1-80.8) | 63.0 (0-73.5) | 0.277 | 0.782 |
| S_5_ | 63.6 (50.0-87.5) | 65.6 (0-78.6) | 0.174 | 0.862 |
| S_6_ | 64.1 (28.2-87.8) | 65.8 (0-81.4) | 0.215 | 0.829 |
| S_7_ | 61.3 (0-87.0) | 63.6 (0-79.0) | 0.205 | 0.837 |
| S_8_ | 79.6 (60.7-96.8) | 80.8 (23.0-90.3) | 0.164 | 0.870 |
| S_9_ | 63.1 (38.0-82.5) | 66.2 (24.6-78.7) | 0.400 | 0.689 |
| S_10_ | 78.2 (0-90.9) | 81.0 (23.9-88.5) | 0.318 | 0.750 |
| S_11_ | 80.0 (55.3-95.6) | 83.6 (20.8-92.3) | 0.174 | 0.862 |
| S_12_ | 74.6 (53.2-100) | 77.4 (24.0-89.3) | 0.267 | 0.790 |

S_1_, S_2_...S_12_ means each CpG site in the region (+729~+834) of *α-internexin*.

GEP-NEN: Gastroenteropancreatic neuroendocrine neoplasm; GI-NEN: Gastrointestinal neuroendocrine neoplasm; pNEN: Pancreatic neuroendocrine neoplasm.
