# Supplementary material for: Loss of expression and prognosis value of alpha-internexin in gastroenteropancreatic neuroendocrine neoplasm
Source: BMC Cancer. 2018 Jun 26;18:691. doi: 10.1186/s12885-018-4449-8 (PMC6020194; doi:10.1186/s12885-018-4449-8)
Supplement: Supplementary file 2 — Table S2. Correlation of α-internexin methylation status with clinicopathological variables. (DOCX 49 kb) [file 12885_2018_4449_MOESM2_ESM.docx]

**Supplementary Table 2** Correlation of *α-internexin* methylation status with clinicopathological variables

| Characteristics | Average of total  12 CpG sites | |  | S_1_ | |  | S_2_ | |  | S_3_ | |  | S_4_ | |  | S_5_ | |  | S6 | |
| --- | --- | --- | --- | --- | --- | --- | --- | --- | --- | --- | --- | --- | --- | --- | --- | --- | --- | --- | --- | --- |
|  | methylation% Median | *P* value |  | methylation% Median | *P* value |  | methylation% Median | *P* value |  | methylation% Median | *P* value |  | methylation% Median | *P* value |  | methylation% Median | *P* value |  | methylation% Median | *P* value |
| **GEP-NENs (n=116)** |  |  |  |  |  |  |  |  |  |  |  |  |  |  |  |  |  |  |  |  |
| Functional status |  | 0.396 |  |  | 0.586 |  |  | 0.319 |  |  | 0.645 |  |  | 0.633 |  |  | 0.297 |  |  | 0.271 |
| Nonfunctional | 65.5 |  |  | 50.0 |  |  | 50.0 |  |  | 50.0 |  |  | 62.8 |  |  | 64.4 |  |  | 626.3 |  |
| Functional | 65.1 |  |  | 48.7 |  |  | 48.3 |  |  | 50.0 |  |  | 62.7 |  |  | 62.5 |  |  | 63.4 |  |
| Tumor location |  | 0.294 |  |  | 0.697 |  |  | 0.569 |  |  | 0.647 |  |  | 0.475 |  |  | 0.203 |  |  | 0.335 |
| Gastrointestinal tract | 65.7 | 0.890 ^d^ |  | 50.0 | 0.770 ^d^ |  | 50.0 | 0.820 ^d^ |  | 50.0 | 0.701 ^d^ |  | 62.7 | 0.918 ^d^ |  | 64.6 | 0.533 ^d^ |  | 66.0 | 0.702 ^d^ |
| Pancreas | 65.1 |  |  | 50.0 |  |  | 50.0 |  |  | 50.0 |  |  | 63.0 |  |  | 63.9 |  |  | 64.9 |  |
| Other | 60.2 |  |  | 46.4 |  |  | 47.6 |  |  | 48.2 |  |  | 60.9 |  |  | 60.4 |  |  | 61.1 |  |
| Tumor grade^a^ |  | 0.611 |  |  | 0.881 |  |  | 0.701 |  |  | 0.808 |  |  | 0.383 |  |  | 0.445 |  |  | 0.192 |
| G1 | 65.6 |  |  | 50.0 |  |  | 50.0 |  |  | 50.0 |  |  | 62.8 |  |  | 64.4 |  |  | 65.1 |  |
| G2 | 63.3 |  |  | 50.0 |  |  | 50.0 |  |  | 50.0 |  |  | 61.2 |  |  | 62.0 |  |  | 61.9 |  |
| G3 | 65.5 |  |  | 50.0 |  |  | 50.0 |  |  | 50.0 |  |  | 63.3 |  |  | 64.4 |  |  | 66.7 |  |
| Tumor type^a^ |  | 0.407 |  |  | 0.632 |  |  | 0.399 |  |  | 0.555 |  |  | 0.171 |  |  | 0.294 |  |  | 0.137 |
| NET | 65.1 |  |  | 50.0 |  |  | 50.0 |  |  | 50.0 |  |  | 62.3 |  |  | 62.7 |  |  | 64.2 |  |
| NEC+MANEC | 65.5 |  |  | 50.0 |  |  | 50.0 |  |  | 50.0 |  |  | 63.3 |  |  | 64.4 |  |  | 66.7 |  |
| Tumor stage |  | 0.350 |  |  | 0.359 |  |  | 0.355 |  |  | 0.414 |  |  | 0.267 |  |  | 0.278 |  |  | 0.419 |
| I+II | 64.4 |  |  | 50.0 |  |  | 49.1 |  |  | 48.6 |  |  | 62.0 |  |  | 63.1 |  |  | 64.2 |  |
| III+IV | 65.7 |  |  | 50.7 |  |  | 50.0 |  |  | 50.0 |  |  | 63.1 |  |  | 64.4 |  |  | 66.7 |  |
| **GI-NENs (n=54)** |  |  |  |  |  |  |  |  |  |  |  |  |  |  |  |  |  |  |  |  |
| Functional status |  | 0.296 |  |  | 0.481 |  |  | 0.481 |  |  | 0.556 |  |  | 0.481 |  |  | 0.481 |  |  | 0.333 |
| Nonfunctional | 65.4 |  |  | 50.0 |  |  | 50.0 |  |  | 50.0 |  |  | 62.8 |  |  | 64.7 |  |  | 66.0 |  |
| Functional | 54.5 |  |  | 41.7 |  |  | 41.1 |  |  | 41.7 |  |  | 55.7 |  |  | 58.2 |  |  | 53.7 |  |
| Tumor grade^b^ |  | 0.081 |  |  | 0.061 |  |  | 0.059 |  |  | 0.056 |  |  | 0.059 |  |  | 0.133 |  |  | 0.067 |
| G1 | 58.9 |  |  | 43.6 |  |  | 42.8 |  |  | 42.9 |  |  | 56.8 |  |  | 60.4 |  |  | 58.0 |  |
| G2 | 65.8 |  |  | 52.2 |  |  | 50.0 |  |  | 50.0 |  |  | 61.7 |  |  | 64.6 |  |  | 65.1 |  |
| G3 | 68.4 |  |  | 54.2 |  |  | 53.1 |  |  | 50.0 |  |  | 64.9 |  |  | 65.0 |  |  | 67.3 |  |
| Tumor type^b^ |  | 0.068 |  |  | 0.122 |  |  | 0.033 |  |  | 0.035 |  |  | 0.036 |  |  | 0.189 |  |  | 0.034 |
| NET | 63.4 |  |  | 50.0 |  |  | 46.2 |  |  | 44.9 |  |  | 60.8 |  |  | 62.5 |  |  | 62.3 |  |
| NEC+MANEC | 68.4 |  |  | 54.2 |  |  | 53.1 |  |  | 50.0 |  |  | 64.9 |  |  | 65.0 |  |  | 67.3 |  |
| Tumor stage |  | 0.016 |  |  | 0.022 |  |  | 0.035 |  |  | 0.023 |  |  | 0.036 |  |  | 0.022 |  |  | 0.062 |
| I+II | 61.7 |  |  | 46.8 |  |  | 46.0 |  |  | 43.4 |  |  | 58.8 |  |  | 62.5 |  |  | 62.3 |  |
| III+IV | 68.4 |  |  | 54.2 |  |  | 53.1 |  |  | 50.0 |  |  | 65.6 |  |  | 66.2 |  |  | 67.5 |  |
| **pNENs (n=49)** |  |  |  |  |  |  |  |  |  |  |  |  |  |  |  |  |  |  |  |  |
| Functional status |  | 0.307 |  |  | 0.703 |  |  | 0.312 |  |  | 0.452 |  |  | 0.541 |  |  | 0.297 |  |  | 0.258 |
| Nonfunctional | 64.7 |  |  | 50.0 |  |  | 50.0 |  |  | 50.0 |  |  | 63.3 |  |  | 65.6 |  |  | 65.4 |  |
| Functional | 65.4 |  |  | 50.0 |  |  | 49.1 |  |  | 50.0 |  |  | 62.8 |  |  | 62.9 |  |  | 63.8 |  |
| Tumor grade^c^ |  | 0.495 |  |  | 0.191 |  |  | 0.268 |  |  | 0.355 |  |  | 0.847 |  |  | 0.585 |  |  | 0.417 |
| G1 | 66.0 |  |  | 51.9 |  |  | 50.0 |  |  | 51.9 |  |  | 63.2 |  |  | 65.6 |  |  | 65.7 |  |
| G2 | 63.3 |  |  | 48.3 |  |  | 50.0 |  |  | 50.0 |  |  | 62.5 |  |  | 62.0 |  |  | 62.5 |  |
| G3 | 62.6 |  |  | 44.3 |  |  | 45.5 |  |  | 44.8 |  |  | 62.9 |  |  | 63.9 |  |  | 63.0 |  |
| Tumor type^c^ |  | 0.293 |  |  | 0.073 |  |  | 0.127 |  |  | 0.155 |  |  | 0.619 |  |  | 0.665 |  |  | 0.357 |
| NET | 65.9 |  |  | 51.4 |  |  | 50.0 |  |  | 50.6 |  |  | 63.1 |  |  | 64.4 |  |  | 65.1 |  |
| NEC+MANEC | 62.6 |  |  | 44.3 |  |  | 45.5 |  |  | 44.8 |  |  | 62.9 |  |  | 63.9 |  |  | 63.0 |  |
| Tumor stage |  | 0.834 |  |  | 0.358 |  |  | 0.967 |  |  | 0.944 |  |  | 0.834 |  |  | 0.967 |  |  | 0.769 |
| I+II | 65.9 |  |  | 50.9 |  |  | 50.0 |  |  | 50.0 |  |  | 63.1 |  |  | 64.4 |  |  | 64.8 |  |
| III+IV | 63.9 |  |  | 48.3 |  |  | 47.6 |  |  | 47.4 |  |  | 62.9 |  |  | 63.9 |  |  | 64.9 |  |

**Supplementary Table 2** Correlation of *α-internexin* methylation status with clinicopathological variables *(Continued)*

| Characteristics | S_7_ | |  | S_8_ | |  | S_9_ | |  | S_10_ | |  | S_11_ | |  | S_12_ | |
| --- | --- | --- | --- | --- | --- | --- | --- | --- | --- | --- | --- | --- | --- | --- | --- | --- | --- |
|  | Methylation% median | *P* value |  | Methylation% median | *P* value |  | Methylation% median | *P* value |  | Methylation% median | *P* value |  | Methylation% median | *P* value |  | Methylation% median | *P* value |
| **GEP-NENs (n=116)** |  |  |  |  |  |  |  |  |  |  |  |  |  |  |  |  |  |
| Functional status |  | 0.374 |  |  | 0.509 |  |  | 0.543 |  |  | 0.626 |  |  | 0.845 |  |  | 0.863 |
| Nonfunctional | 62.0 |  |  | 82.1 |  |  | 64.4 |  |  | 78.9 |  |  | 81.3 |  |  | 75.0 |  |
| Functional | 61.5 |  |  | 80.0 |  |  | 62.7 |  |  | 77.8 |  |  | 79.3 |  |  | 75.4 |  |
| Tumor location |  | 0.181 |  |  | 0.326 |  |  | 0.071 |  |  | 0.113 |  |  | 0.139 |  |  | 0.099 |
| Gastrointestinal tract | 62.6 | 0.682 ^d^ |  | 82.4 | 0.871 ^d^ |  | 65.1 | 0.945 ^d^ |  | 80.0 | 0.792 ^d^ |  | 82.1 | 0.741 ^d^ |  | 76.5 | 0.926 ^d^ |
| Pancreas | 61.7 |  |  | 80.0 |  |  | 63.8 |  |  | 78.4 |  |  | 80.3 |  |  | 75.4 |  |
| Other | 59.3 |  |  | 76.9 |  |  | 57.5 |  |  | 75.0 |  |  | 77.9 |  |  | 71.1 |  |
| Tumor grade^a^ |  | 0.427 |  |  | 0.322 |  |  | 0.431 |  |  | 0.454 |  |  | 0.867 |  |  | 0.619 |
| G1 | 62.2 |  |  | 79.8 |  |  | 63.8 |  |  | 78.2 |  |  | 79.0 |  |  | 75.4 |  |
| G2 | 61.1 |  |  | 78.7 |  |  | 63.3 |  |  | 76.4 |  |  | 80.3 |  |  | 73.7 |  |
| G3 | 62.5 |  |  | 82.4 |  |  | 64.5 |  |  | 79.4 |  |  | 82.1 |  |  | 75.9 |  |
| Tumor type^a^ |  | 0.344 |  |  | 0.204 |  |  | 0.378 |  |  | 0.373 |  |  | 0.595 |  |  | 0.501 |
| NET | 61.7 |  |  | 79.6 |  |  | 63.3 |  |  | 78.0 |  |  | 79.3 |  |  | 75.0 |  |
| NEC+MANEC | 62.5 |  |  | 82.4 |  |  | 64.5 |  |  | 79.4 |  |  | 82.1 |  |  | 75.9 |  |
| Tumor stage |  | 0.927 |  |  | 0.873 |  |  | 0.971 |  |  | 0.465 |  |  | 0.746 |  |  | 0.886 |
| I+II | 61.7 |  |  | 79.8 |  |  | 63.8 |  |  | 77.8 |  |  | 79.8 |  |  | 74.6 |  |
| III+IV | 62.2 |  |  | 82.4 |  |  | 64.5 |  |  | 79.5 |  |  | 82.1 |  |  | 75.8 |  |
| **GI-NENs (n=54)** |  |  |  |  |  |  |  |  |  |  |  |  |  |  |  |  |  |
| Functional status |  | 0.259 |  |  | 0.370 |  |  | 0.333 |  |  | 0.370 |  |  | 0.296 |  |  | 0.259 |
| Nonfunctional | 62.7 |  |  | 82.4 |  |  | 65.1 |  |  | 80.0 |  |  | 82.1 |  |  | 76.6 |  |
| Functional | 54.2 |  |  | 67.5 |  |  | 50.0 |  |  | 67.4 |  |  | 63.6 |  |  | 59.3 |  |
| Tumor grade^b^ |  | 0.221 |  |  | 0.033 |  |  | 0.130 |  |  | 0.084 |  |  | 0.090 |  |  | 0.226 |
| G1 | 58.3 |  |  | 72.8 |  |  | 57.5 |  |  | 71.8 |  |  | 70.8 |  |  | 67.5 |  |
| G2 | 62.0 |  |  | 79.6 |  |  | 63.3 |  |  | 81.3 |  |  | 82.8 |  |  | 77.8 |  |
| G3 | 65.4 |  |  | 83.0 |  |  | 65.8 |  |  | 80.4 |  |  | 82.8 |  |  | 76.8 |  |
| Tumor type^b^ |  | 0.139 |  |  | 0.014 |  |  | 0.057 |  |  | 0.060 |  |  | 0.113 |  |  | 0.142 |
| NET | 62.0 |  |  | 78.0 |  |  | 63.3 |  |  | 76.3 |  |  | 78.5 |  |  | 75.8 |  |
| NEC+MANEC | 65.4 |  |  | 83.0 |  |  | 65.8 |  |  | 80.4 |  |  | 82.8 |  |  | 76.8 |  |
| Tumor stage |  | 0.137 |  |  | 0.192 |  |  | 0.107 |  |  | 0.018 |  |  | 0.044 |  |  | 0.080 |
| I+II | 61.5 |  |  | 79.2 |  |  | 62.7 |  |  | 72.7 |  |  | 77.9 |  |  | 71.4 |  |
| III+IV | 65.4 |  |  | 83.0 |  |  | 65.3 |  |  | 80.9 |  |  | 83.3 |  |  | 78.4 |  |
| **pNENs (n=49)** |  |  |  |  |  |  |  |  |  |  |  |  |  |  |  |  |  |
| Functional status |  | 0.417 |  |  | 0.383 |  |  | 0.336 |  |  | 0.307 |  |  | 0.535 |  |  | 0.810 |
| Nonfunctional | 61.8 |  |  | 79.6 |  |  | 64.0 |  |  | 78.9 |  |  | 80.0 |  |  | 73.7 |  |
| Functional | 61.6 |  |  | 80.4 |  |  | 63.0 |  |  | 78.1 |  |  | 80.0 |  |  | 75.4 |  |
| Tumor grade^c^ |  | 0.448 |  |  | 0.663 |  |  | 0.461 |  |  | 0.631 |  |  | 0.584 |  |  | 0.399 |
| G1 | 62.9 |  |  | 80.8 |  |  | 63.8 |  |  | 79.0 |  |  | 81.0 |  |  | 75.4 |  |
| G2 | 61.3 |  |  | 79.3 |  |  | 63.5 |  |  | 78.4 |  |  | 83.0 |  |  | 76.2 |  |
| G3 | 60.9 |  |  | 76.8 |  |  | 60.9 |  |  | 77.2 |  |  | 78.9 |  |  | 70.3 |  |
| Tumor type^c^ |  | 0.278 |  |  | 0.411 |  |  | 0.236 |  |  | 0.411 |  |  | 0.341 |  |  | 0.187 |
| NET | 61.9 |  |  | 80.4 |  |  | 63.8 |  |  | 78.7 |  |  | 81.7 |  |  | 75.8 |  |
| NEC+MANEC | 60.9 |  |  | 76.8 |  |  | 60.9 |  |  | 77.2 |  |  | 78.9 |  |  | 70.3 |  |
| Tumor stage |  | 0.304 |  |  | 0.856 |  |  | 0.304 |  |  | 0.944 |  |  | 0.706 |  |  | 0.361 |
| I+II | 61.9 |  |  | 80.4 |  |  | 63.8 |  |  | 78.4 |  |  | 81.7 |  |  | 75.8 |  |
| III+IV | 60.9 |  |  | 78.7 |  |  | 60.9 |  |  | 78.9 |  |  | 78.9 |  |  | 71.4 |  |

S_1_, S_2_...S_12_ means each CpG site in the region (+729~+834) of *α-internexin*. ^a^ 113 cases both for tumor grade and tumor type; ^b^ 52 cases both for tumor grade and tumor type;

^c^ 48cases both for tumor grade and tumor type. ^d^ The *P* value were computed by the contrast between gastrointestinal tract and pancreas.

GEP-NEN: Gastroenteropancreatic neuroendocrine neoplasm; NET: Neuroendocrine tumor; NEC: Neuroendocrine carcinoma; MANEC: Mixed adenoneuroendocrine carcinoma; GI-NEN: Gastrointestinal neuroendocrine neoplasm; pNEN: Pancreatic neuroendocrine neoplasm.
