# Supplementary material for: Loss of expression and prognosis value of alpha-internexin in gastroenteropancreatic neuroendocrine neoplasm
Source: BMC Cancer. 2018 Jun 26;18:691. doi: 10.1186/s12885-018-4449-8 (PMC6020194; doi:10.1186/s12885-018-4449-8)
Supplement: Supplementary file 3 — Table S3. Correlation of α-internexin methylation status with overall survival. (DOCX 28 kb) [file 12885_2018_4449_MOESM3_ESM.docx]

**Supplementary Table 3** Correlation of *α-internexin* methylation status with overall survival

|  | GEP-NENs (n=116) | | | |  | GI-NENs (n=54) | | | |  | pNENs (n=49) | | | |
| --- | --- | --- | --- | --- | --- | --- | --- | --- | --- | --- | --- | --- | --- | --- |
|  | Overall survival, mean (years) | | χ2 value | *P* value |  | Overall survival, mean (years) | | χ2 value | *P* value |  | Overall survival, mean (years) | | χ2 value | *P* value |
|  | Methylation% ≥50% | Methylation% <50% |  |  |  | Methylation% ≥68% | Methylation% <68% |  |  |  | Methylation% ≥48% | Methylation% <48% |  |  |
| Average of total 12 CpG sites | 3.7 | 8.2 | 1.540 | 0.215 |  | 4.5 | 7.0 | 0.221 | 0.638 |  | 3.8 | 5.6 | 0.561 | 0.454 |
| S_1_ | 5.0 | 8.3 | 0.030 | 0.863 |  | 1.2 | 7.4 | 2.647 | 0.104 |  | 5.7 | 5.4 | 0.426 | 0.514 |
| S_2_ | 4.9 | 8.5 | 0.103 | 0.748 |  | 3.0 | 7.2 | 0.436 | 0.509 |  | 5.0 | 5.9 | 2.224 | 0.136 |
| S_3_ | 5.1 | 8.3 | 0.006 | 0.937 |  | — | 7.2 | NC^a^ | NC^a^ |  | 5.0 | 6.0 | 2.564 | 0.109 |
| S_4_ | 3.4 | 8.3 | 0.129 | 0.719 |  | 2.8 | 7.2 | 0.012 | 0.912 |  | 2.8 | 5.7 | 0.232 | 0.630 |
| S_5_ | 3.7 | 8.2 | 1.263 | 0.261 |  | 4.4 | 7.1 | 0.073 | 0.787 |  | 3.8 | 5.6 | 0.561 | 0.454 |
| S_6_ | 3.7 | 8.1 | 2.134 | 0.144 |  | 4.6 | 6.8 | 0.653 | 0.419 |  | 5.5 | 4.0 | 0.864 | 0.353 |
| S_7_ | 3.7 | 8.2 | 1.540 | 0.215 |  | 5.0 | 6.7 | 1.202 | 0.273 |  | 3.8 | 5.6 | 0.561 | 0.454 |
| S_8_ | 3.6 | 8.2 | 0.999 | 0.318 |  | 3.6 | 6.7 | 2.551 | 0.110 |  | 3.7 | 5.6 | 0.289 | 0.591 |
| S_9_ | 3.6 | 8.3 | 0.754 | 0.385 |  | 4.5 | 7.0 | 0.331 | 0.565 |  | 5.6 | 3.9 | 0.709 | 0.400 |
| S_10_ | 3.5 | 8.3 | 0.328 | 0.567 |  | 3.8 | 6.9 | 1.130 | 0.288 |  | 5.6 | 4.0 | 0.422 | 0.516 |
| S_11_ | 3.6 | 8.3 | 0.752 | 0.386 |  | 3.1 | 7.1 | 0.093 | 0.760 |  | 3.7 | 5.6 | 0.289 | 0.591 |
| S_12_ | 3.6 | 8.3 | 0.752 | 0.386 |  | 3.4 | 7.0 | 0.295 | 0.587 |  | 3.7 | 5.6 | 0.289 | 0.591 |

S_1_, S_2_...S_12_ means each CpG site in the region (+729~+834) of *α-internexin*. ^a^ None of the patients had methylation% ≥68% so that the statistical calculation wasn’t performed.

GEP-NEN: Gastroenteropancreatic neuroendocrine neoplasm; GI-NEN: Gastrointestinal neuroendocrine neoplasm; pNEN: Pancreatic neuroendocrine neoplasm. NC, Not computable.
